# Behavioral Analysis of Postgraduate Education Satisfaction: Unveiling Key Influencing Factors with Bayesian Networks and Feature Importance

**DOI:** 10.3390/bs15040559

**Published:** 2025-04-21

**Authors:** Sheng Li, Ting Wang, Hanqing Yin, Shuai Ding, Zhiqiang Cai

**Affiliations:** 1 Graduate School, Northwestern Polytechnical University, Xi’an 710072, China; lisheng@nwpu.edu.cn (S.L.); yinhq@nwpu.edu.cn (H.Y.); 2 Department of Industrial Engineering, Northwestern Polytechnical University, Xi’an 710072, China; wt3377@mail.nwpu.edu.cn; 3 School of Public Policy and Administration, Northwestern Polytechnical University, Xi’an 710072, China; dingshuai@mail.nwpu.edu.cn

**Keywords:** postgraduate education satisfaction, two-stage feature optimization, Bayesian network, Birnbaum importance, behavioral analysis

## Abstract

Accurately evaluating postgraduate education satisfaction is crucial for improving higher education quality and optimizing management practices. Traditional methods often fail to capture the complex behavioral interactions among influencing factors. In this study, an innovative satisfaction indicator system framework is proposed that integrates a two-stage feature optimization method and the Tree Augmented Naive Bayes (TAN) model. The framework is designed to assess key satisfaction drivers across seven dimensions: course quality, research projects, mentor guidance, mentor’s role, faculty management, academic enhancement, and quality development. Using data from 8903 valid responses, Confirmatory Factor Analysis (CFA) was conducted to validate the framework’s reliability. The two-stage feature optimization method, including statistical pre-screening and XGBoost-based recursive feature selection, refined 49 features to 29 core indicators. The TAN model was used to construct a causal network, revealing the dynamic relationships between factors shaping satisfaction. The model outperformed four common machine learning algorithms, achieving an AUC value of 91.01%. The Birnbaum importance metric was employed to quantify the contribution of each feature, revealing the critical roles of academic resilience, academic aspirations, dedication and service spirit, creative ability, academic standards, and independent academic research ability. This study offers management recommendations, including enhancing academic support, mentorship, and interdisciplinary learning. Its findings provide data-driven insights for optimizing key indicators and improving postgraduate education satisfaction, contributing to behavioral sciences by linking satisfaction to outcomes and practices.

## 1. Introduction

Currently, the nation is actively advancing a high-quality education system, focusing on cultivating top innovative talents. This effort strengthens the foundation of education, science, and talent, essential for building a modernized socialist nation and enhancing the training of high-level professionals. Postgraduate education, as the highest level of education, plays a vital role in enhancing national competitiveness. Improving its quality is essential not only for universities to remain competitive but also for the broader development of the nation’s educational system. Postgraduate satisfaction, as a key indicator of educational quality, has become a focal point of attention. Satisfaction surveys provide valuable insights into students’ perceptions of various aspects such as curriculum design, teaching quality, and available resources, all of which are shaped by behavioral and psychological factors. These insights help administrators identify areas that need improvement, shape educational policies, and allocate resources in ways that are more aligned with students’ behavioral responses and their academic experiences.

Despite extensive research on educational satisfaction, significant limitations remain. Many studies rely on traditional statistical methods, such as surveys and correlation analyses, which may not fully capture the complex behavioral interactions among satisfaction-driving factors. Additionally, some methods depend on linear assumptions or subjective weighting, which can limit their ability to account for the behavioral complexity of educational systems. Additionally, existing research has not sufficiently addressed the dynamic, causal relationships between behavioral factors and satisfaction, nor has it effectively quantified the importance of individual features. This research gap is exacerbated by the underutilization of advanced artificial intelligence techniques, such as Bayesian networks, which can provide more comprehensive and robust solutions by uncovering underlying behavioral patterns.

To overcome these challenges, a new framework is introduced in this study to evaluate postgraduate education satisfaction, integrating behavioral insights. The framework uses a two-step feature optimization process, including statistical pre-screening and XGBoost-based feature elimination, to select the most important factors. A Tree Augmented Naive Bayes (TAN) model is used to build a causal network, revealing how these factors interact. The model’s reliability is confirmed through confusion matrices and performance metrics. Additionally, Birnbaum importance metrics are used to quantify the marginal contributions of individual features to satisfaction, providing deep behavioral insights into the critical roles of academic enhancement, quality development, and the mentor’s role. This approach not only enhances the interpretability and scientific rigor of satisfaction evaluations but also provides actionable, data-driven recommendations for improving key behavioral indicators in postgraduate education.

By integrating behavioral analysis with the two-stage feature optimization method and Bayesian networks, this study overcomes the limitations of traditional statistical approaches, offering an innovative solution for causal modeling and feature importance quantification. The findings will contribute to both theoretical advancements and practical applications, providing new pathways for improving education quality and ensuring the effectiveness of educational policies.

## 2. Literature Review

### 2.1. Measurement and Key Factors of Postgraduate Education Satisfaction

Postgraduate education satisfaction, as a critical indicator of higher education quality, is typically defined as students’ subjective evaluation of their overall learning experience, based on a comparison between their expectations and actual perceptions of educational services (Muijs & Bokhove, 2017). Existing research commonly employs survey questionnaires and multidimensional models to measure satisfaction, with core indicators encompassing course quality, mentor support, learning environment, and career development opportunities. However, these factors do not function in isolation; they interact with students’ cognitive, emotional, and behavioral responses, influencing their overall satisfaction.

The literature reveals the diverse and complex nature of influencing factors:

The first is physical and environmental factors. The quality of indoor environmental conditions significantly impacts postgraduate students’ learning comfort and satisfaction, and key factors such as thermal comfort, air quality, lighting, and noise levels are highlighted. Optimizing the design of learning spaces has been shown to effectively enhance satisfaction and learning efficiency (Al-Dmour, 2024). The second is career development and educational support. The effective use of career-related social networks (e.g., LinkedIn) and social media not only contributes to learning interactions but also plays a crucial role in improving career development prospects and satisfaction (Hazzam et al., 2024). The third set of factors are teaching and curriculum design. The modernization of course content and the integration of advanced technologies, such as 3D printing methods, have significantly enhanced postgraduate students’ learning experiences, thereby boosting their satisfaction (Allanqawi et al., 2023). The final factors are disciplinary and regional differences. Significant variations in satisfaction levels exist across different disciplines and geographic regions. For instance, fields with a strong clinical or applied focus report higher satisfaction levels due to clear career development pathways and practical skills training (Bush & Lowery, 2016).

In summary, postgraduate education satisfaction not only influences students’ academic performance but is also closely associated with their career success (Pérez Fuentes et al., 2023).

### 2.2. Methods for Postgraduate Education Satisfaction Evaluation

The evaluation process typically involves indicator weighting and data modeling.

In determining indicator weights, subjective weighting methods (e.g., analytic hierarchy process) rely on expert judgment, which introduces an inherent level of subjectivity. In contrast, objective weighting methods (e.g., entropy weight) are based on the characteristics of the data but may overlook the practical significance of indicators (Guo & Li, 2018). Recently, hybrid weighting approaches that combine subjective and objective methods have garnered attention, which aim to balance practical significance and result stability; however, they often face increased complexity during the integration process.

In terms of data modeling, traditional linear models (e.g., multiple regression and gray system models) perform well with small sample sizes but struggle to capture the nonlinear relationships inherent in satisfaction (Sharif et al., 2012). Meanwhile, modern machine learning methods (e.g., BP neural networks) excel in high-dimensional data analysis, demonstrating superior performance. However, these methods are prone to overfitting and often lack interpretability. In recent years, hybrid models have emerged as a mainstream approach. For instance, the combination of BP with a gray system leverages their complementary strengths, effectively handling both nonlinear relationships and sparse data (Tanveer et al., 2023).

Bayesian networks (BNs) have shown promise as a potential tool for postgraduate education satisfaction research due to their ability to model causal relationships and dynamically update predictions. Unlike traditional models, BNs can capture the behavioral dependencies among various satisfaction-related factors, offering a deeper understanding of how students’ cognitive and emotional responses influence their overall educational experience. Furthermore, they support real-time updates with dynamic data, enabling the long-term monitoring and optimization of satisfaction levels (R. Huang et al., 2014).

However, the application of BNs in the educational field faces several challenges. First, the construction of accurate models relies heavily on high-quality data and prior knowledge. Second, in scenarios involving a large number of variables, the computational resource demands can be substantial. Lastly, inferring causal relationships requires the integration of domain expertise and experimental validation to avoid overfitting and ensure the robustness of the conclusions (Weber et al., 2012).

### 2.3. Limitations of Existing Studies

Despite the progress made, there are still four notable limitations in research:

(1) Insufficient exploration of causal relationships: traditional statistical methods struggle to reveal complex causal relationships and fail to comprehensively analyze the interactions among multidimensional factors.
(2) A lack of multidimensional integrated analysis: existing studies rarely consider the combined effects of factors such as the environment and curriculum on satisfaction. This omission limits the understanding of how these crucial dimensions interact and influence satisfaction (Y. Zhang et al., 2024).
(3) Insufficient model applicability and interpretability: modern machine learning methods, such as Support Vector Machines (SVMs) and neural networks, perform well in predictions. However, their “black-box” nature limits the interpretability of research findings and reduces their practical applicability as guidance for decision-making.
(4) Limitations of feature selection techniques: many studies rely on subjective weighting or simple statistical methods for feature importance evaluation. These approaches lack rigorous and scientific quantitative foundations, which could lead to the omission of key variables.

This study contributes to global scholarship in three key areas: cross-cultural studies of graduate student satisfaction, policy-oriented research on higher education quality assurance, and behavioral modeling in institutional assessment frameworks:

(1) Our findings align with international research on graduate satisfaction, particularly studies that explore satisfaction differences between online and blended doctoral programs (Erichsen et al., 2014). Similarly, research highlights how cultural and contextual factors, such as nationality, influence student satisfaction (Stewart et al., 2018), emphasizing the need to consider diverse satisfaction drivers (Crede & Borrego, 2014).
(2) This study also engages with global discussions on educational policies. For instance, research has examined how academic dismissal policies impact dropout rates and satisfaction (Sneyers & De Witte, 2017). Our study emphasizes the critical role of academic support and mentorship, advocating for policies that promote holistic student experiences—similar to the policy issues surrounding international student housing (Ramia et al., 2022).
(3) By utilizing Bayesian networks and behavioral modeling, this research advances the use of data-driven methods in higher education assessment. It builds on existing work that connects supervisory styles to student creativity through psychological factors (Walker & Palmer, 2011). Furthermore, it contributes to studies on the impact of student funding policies (Czarnecki & Litwiński, 2024), illustrating how behavioral models can offer deeper insights into educational practices (Gu et al., 2017).

## 3. Methods

### 3.1. Analysis of Questionnaire and Indicator System

To ensure the reliability and validity of the questionnaire, a systematic indicator system was developed, drawing on extensive literature and expert reviews. Seven core dimensions were identified: course quality, research projects, mentor guidance, mentor’s role, faculty management, academic enhancement, and quality development, each with corresponding indicators. CFA was used to assess the structural rationality and consistency of the indicators.

Reliability analysis was conducted by calculating Cronbach’s α (with values above 0.7 considered satisfactory) and Composite Reliability (CR), evaluating the internal consistency of the questionnaire and its dimensions. A validity analysis was carried out using Average Variance Extracted (AVE) to assess the explanatory power of latent variables for the observed variables (X. Zhang et al., 2023). The formula is as follows:

$$AVE=\frac{\sum_{i=1}^{n}\lambda_{i}^{2}}{\sum_{i=1}^{n}\lambda_{i}^{2}+\sum_{i=1}^{n}\theta_{i}},i=1,2,\ldots ,n$$

where n is the number of features, i denotes a specific feature, $\lambda_{i}$ represents the standardized factor loadings, and $\theta_{i}$ denotes the error terms.

CFA was used to test the goodness-of-fit between the hypothesized factor structure and the actual data. Additionally, the Kaiser–Meyer–Olkin (KMO) test and Bartlett’s test of sphericity were employed to verify the suitability of the factor analysis, ensuring that the questionnaire design demonstrated robust scientific validity and reliability.

### 3.2. Two-Stage Feature Optimization Method

To identify the most representative features from the initial set of 49 for inclusion in the causal network modeling process, a two-stage feature optimization method with the following steps is proposed:

Stage One: Statistical Feature Pre-Screening

To enhance the efficiency of feature selection, this study employed three statistical methods: Variance Thresholding (VT), Correlation Analysis (CA), and Mutual Information (MI). Features with variances below the threshold were eliminated. Subsequently, using CA coefficients combined with MI values, features exhibiting minimal correlation or limited contribution to the target variable were excluded.

To ensure robustness in the screening criteria, fixed thresholds were replaced with data-driven dynamic thresholds. Features were retained when they simultaneously satisfied the following conditions:

(1) Features with variance higher than the 15th percentile of the dataset’s variance distribution (≥0.61).
(2) Features with an absolute correlation coefficient greater than the 15th percentile of the dataset’s correlation distribution with the target variable (|Corr|≥0.43) and feature-to-feature correlation matrix values of |Corr|≤0.95 to reduce multicollinearity.
(3) Features with mutual information values greater than the 15th percentile of the dataset (≥0.17).

Stage Two: XGBoost-Based Recursive Feature Elimination (RFE)

XGBoost was chosen as the base model for RFE due to its high performance and efficiency through parallel computation and regularization optimization. It effectively handles high-dimensional nonlinear data and provides clear feature importance evaluation, forming a solid foundation for recursive feature selection. In this stage, feature importance is initially assessed using XGBoost’s built-in method, ranking features based on their gain values (J. Chen et al., 2020).

The calculation formula is as follows:

$$Gain(T,i)=\frac{1}{\left|T\right|}\sum_{t\in T}Gain(t,i)$$

where T represents the set of all trees and Gain(T,i) refers to the gain achieved by feature i during its split within tree t.

Subsequently, RFE combined with cross-validation was employed to iteratively remove features with the lowest gain, thereby optimizing the feature subset (Q. Chen et al., 2018). The ultimate objective is to maximize contribution while minimizing redundancy.

The formula for the feature scoring function is as follows:

$$Score(i)=\sum_{i=1}^{n}\beta_{i}^{2}$$

where $\beta_{i}$ represents the regression coefficient of the i-th feature in the model, and n denotes the total number of features.

In addition, to validate the effectiveness, the ReliefF and PSO methods were included in a comparative experiment. This multi-faceted evaluation of different strategies ensures that the final selected feature subset demonstrates superior practicality and stability.

### 3.3. Bayesian Network Theory

Based on probabilistic graphical models, a Bayesian network (BN) represents the conditional dependencies and joint probability distributions among random variables. The core concept derives from Bayes’ theorem, which combines prior and conditional probabilities to infer posterior probabilities, allowing for dynamic belief updates in a hypothesis (Si et al., 2019). The formula is as follows:

$$P(A|B)=\frac{P(B|A)\cdot P(A)}{P(B)}$$

where P(A|B) represents the posterior probability of event A given that event B has occurred; and P(B|A) is the conditional probability of B given that A has occurred. P(A) and P(B) are the prior and marginal probabilities of events A and B, respectively.

Naive Bayes (NB) applies Bayes’ theorem with the assumption of feature independence, simplifying computational complexity. The Tree-Augmented Naive Bayes (TAN) model enhances this by introducing dependencies between features through a tree structure. TAN maximizes the likelihood function to optimize the feature dependencies (Xiao et al., 2022). The formula is expressed as follows:

$$P\left(C|A\right)\propto P\left(C\right)\cdot \prod_{i=1}^{n}P(A_{i}|Parents(A_{i}))$$

where C represents the class, ${A=\{A}_{1},A_{2},\ldots ,A_{n}\}$ denotes the set of n feature variables, and $parents(A_{i})$ refers to the parent node of feature $A_{i}$.

**Figure 1:**
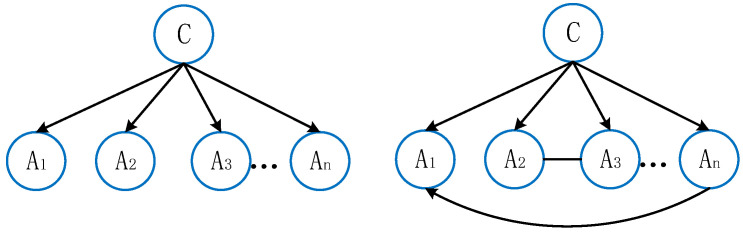
NB and TAN structures.

Figure 1 illustrates examples of NB and TAN structures.

Compared to fully connected Bayesian networks, TAN significantly reduces computational complexity by constructing a maximum weight spanning tree while retaining dependency information among features. The modeling process is shown in Table 1.

This study evaluates the performance of the classifier using a confusion matrix (Y. Wang et al., 2020). The commonly used metrics and their specific formulas are as follows:

Accuracy (Acc) denotes the ratio of correctly classified samples to the total number of samples, which is the sum of True Positive (*TP*), True Negative (*TN*), False Positive (*FP*), and False Negative (*FN*):

$$Acc=\frac{TP+TN}{TP+TN+FP+FN}$$

Recall (R) denotes the ratio of actual positive samples that are correctly identified:

$$R=\frac{TP}{TP+FN}$$

Precision (P) denotes the ratio of predicted positive samples that are truly positive:

$$P=\frac{TP}{TP+FP}$$

F1-Score (F1) is the harmonic mean of precision and recall, considering both metrics:

$$F1=\frac{2*Recall*Precision}{Recall+Precision}$$

True Positive Rate (TPR) and False Positive Rate (FPR) are defined as follows:

$$TPR=\frac{TP}{TP+FN},\;\;FPR=\frac{FP}{TN+FP}$$

The ROC curve plots True Positive Rate (TPR) against False Positive Rate (FPR) to evaluate model performance across different classification thresholds, with the Area Under the Curve (AUC) serving as a key performance indicator. In multi-class tasks, the “one vs. all” approach is used, and in this study, five ROC curves were calculated to assess the model’s ability to distinguish between categories.

**Figure 2:**
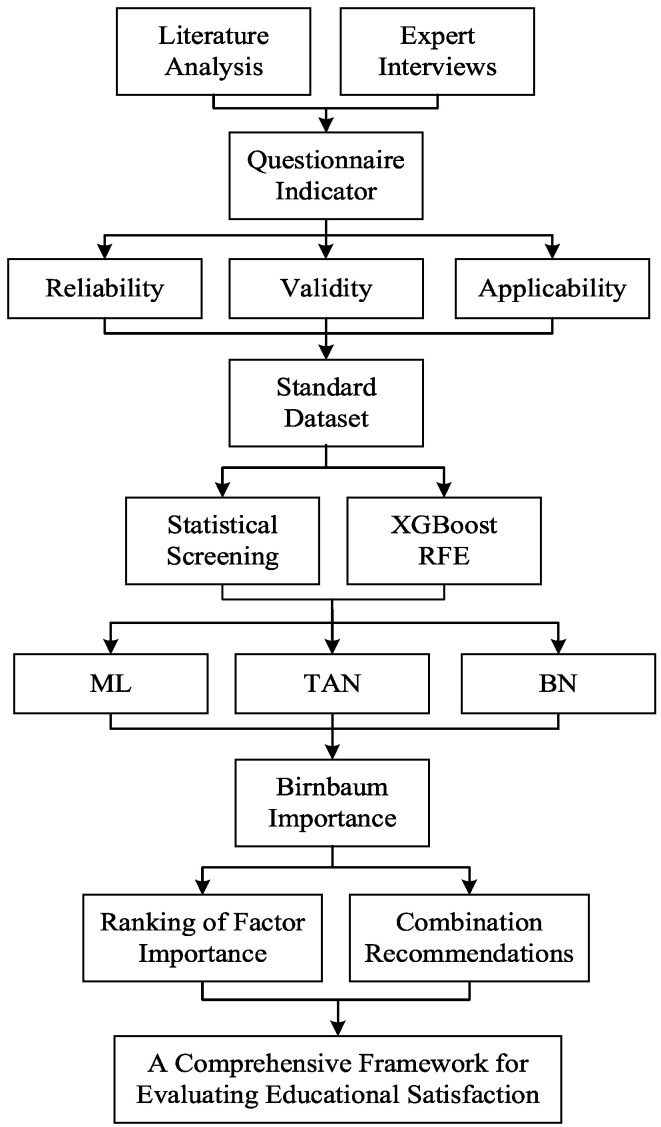
The experimental flow.

The experimental flow of this study is shown in Figure 2.

### 3.4. Importance Measurement Method

The theory of importance measurement, a key area in reliability mathematics, analyzes the contribution of individual components to overall system performance. This study uses the Birnbaum importance metric to quantitatively assess the marginal contributions of satisfaction indicators to overall satisfaction. Based on probabilistic inference, Birnbaum evaluates feature importance by comparing the impact of features on the posterior probability distribution of the target variable, focusing on system reliability sensitivity to individual feature states (Hu et al., 2017), expressed by the following formula:

$$I^{B}\left(i|t\right)=\frac{\partial h\left(t\right)}{\partial p_{i}\left(t\right)},i=1,2,\ldots ,n$$

where $h\left(t\right)$ represents the system reliability, and $p_{i}\left(t\right)$ denotes the state probability of the i-th feature.

In this study, all indicators adopt a five-point Likert scale (ranging from 1 to 5), indicating that each variable has five discrete states. To accommodate satisfaction evaluation with multistate features, importance measurement theory was extended from binary systems to multistate systems (Dang et al., 2023). The formula is as follows:

$${I\left(BM\right)}_{C_{i}}^{S}=\frac{1}{w_{i}-1}\sum_{j=1}^{w_{i}}\left|P\left(S=5|C_{i}=j\right)-P\left(S=5\right)\right|$$

where S represents the target variable, namely, the predicted outcome of satisfaction; $C_{i}$ denotes a feature, with each variable having w possible states (corresponding to the Likert scale, w=5); P(S=5) refers to the prior probability of the target variable; and $P\left(S=5|C_{i}=j\right)$ is the posterior probability given $C_{i}=j$.

This study applied importance measurement theory to the five-point Likert scale, extending traditional binary systems to more precisely quantify the marginal contributions of features across multistate settings (Song et al., 2024). An in-depth analysis of the seven critical dimensions was conducted to calculate each dimension’s contribution to overall satisfaction under different states.

## 4. Data Processing and Feature Selection

### 4.1. Satisfaction Score Dataset

Indicator system overview: the final questionnaire encompasses seven primary dimensions, including course quality (M. Wang & Wu, 2019), research projects (Belash et al., 2015), mentor guidance (Sheng et al., 2024), mentor’s role (Avramkova et al., 2021), faculty management (H. Zhang et al., 2022), academic enhancement (Pineda, 2013), and quality development (J. Zhang et al., 2015). These dimensions are further subdivided into 49 secondary indicators, resulting in a total of 49 feature variables (labeled $X_{1}$ to $X_{49}$), with overall educational satisfaction (Y) serving as the overarching evaluation variable.

The detailed indicator structure is presented in Table 2.

Data source: The dataset used in this study was derived from a satisfaction survey conducted among all postgraduate students at the university. A total of 9122 samples were collected. The questionnaire was designed based on a five-point Likert scale, encompassing 49 features and one target. The variable values range from 1 to 5, corresponding to five levels of satisfaction from “Very Dissatisfied” to “Very Satisfied”.

Data preprocessing: After data collection, a thorough quality check was conducted. Specifically, samples with missing values and redundant entries were excluded. It was ensured that all variable values remained within the range of 1 to 5, and any outliers were eliminated. After these steps, 8903 valid samples were retained, accounting for 97.6% of the total dataset.

### 4.2. Reliability and Validity Analysis of the Questionnaire

CFA was employed to validate the structural rationality of the questionnaire. Each dimension was analyzed individually, and when a dimension exhibited low value, further examination was conducted to identify problematic indicators. The results, as shown in Table 3, indicate that the questionnaire on educational satisfaction demonstrated good performance overall, though it varied across different dimensions.

Reliability performance: With the exception of the research projects dimension, the Cronbach’s α and CR values for all other dimensions exceeded 0.8, indicating strong internal consistency across the questionnaire as a whole. Key dimensions such as mentor guidance, mentor’s role, academic enhancement, and quality development demonstrated particularly outstanding reliability.

Validity performance: The AVE values for all dimensions surpassed 0.5, signifying that latent factors exhibited strong explanatory power over observed variables. Notably, the dimensions mentor guidance and mentor’s role achieved AVE values exceeding 0.85, reflecting well-designed constructs and strong consistency among indicators.

Applicability performance: KMO values for all dimensions exceeded 0.5, with most surpassing 0.9, confirming that the questionnaire data were well-suited for factor analysis. Additionally, Bartlett’s sphericity test yielded significant results across all dimensions (p<0.001), further validating the suitability.

Overall, the questionnaire achieved a Cronbach’s α of 0.9755 and a KMO value of 0.9823, demonstrating high rationality in its overall design. These results affirm its suitability as a comprehensive tool for evaluating postgraduate education satisfaction.

### 4.3. Feature Selection for the Evaluation System

To enhance the accuracy of the educational satisfaction evaluation, a two-stage feature optimization method was adopted to screen the initial 49 features.

Stage 1: Statistical Feature Pre-Screening

This stage integrates VT, CA, and MI analyses, providing an assessment of both the redundancy between features and the correlation with Y.

After the screening process, 33 features were retained from the original 49. In Stage 1, the excluded features primarily came from the following dimensions: course quality ($X_{1}$, $X_{2}$), research projects ($X_{9}$, $X_{10}$), mentor guidance ($X_{11}$, $X_{12}$, $X_{13}$, $X_{15}$, $X_{16}$), mentor’s role ($X_{20}$, $X_{21}$), and faculty management ($X_{27}$, $X_{28}$, $X_{29}$, $X_{30}$, $X_{31}$). Regarding reliability, the academic enhancement and quality development dimensions demonstrated particularly strong internal consistency, as reflected by their Cronbach’s α values of 0.9709 and 0.9772, respectively. In contrast, features from dimensions like course quality and research projects were less reliable (Cronbach’s α values of 0.9096 and 0.7924), which further justified the exclusion of certain features from these dimensions.

By replacing fixed thresholds with dynamic thresholds, this method adapts to the characteristics of the data distribution. This ensures that the selected features contribute significantly to the target variable while minimizing redundancy.

Stage 2: XGBoost Recursive Feature Elimination

The 33 features filtered in the first stage were used as input to construct an XGBoost classifier. The model’s built-in feature importance evaluation method was employed to calculate the contribution of each feature to the target based on their gain values.

The RFE process followed these steps: first, based on importance rankings, the feature with the lowest contribution was identified and removed, and the feature set was updated accordingly. Then, five-fold cross-validation was used to evaluate the model’s performance (Acc) after each removal. If there was no significant drop in the Acc metrics, the process continued iteratively until it stabilized. This determined the optimal subset.

Through five-fold cross-validation and parameter tuning, the optimal hyperparameters for the XGBoost model were determined as follows: a learning rate of 0.2, a max_depth of 3, and n_estimators set to 50. The second stage further refined the feature set by removing features such as $X_{6}$, $X_{7}$, $X_{19}$, and $X_{33}$, based on their contribution to model performance.

**Figure 3:**
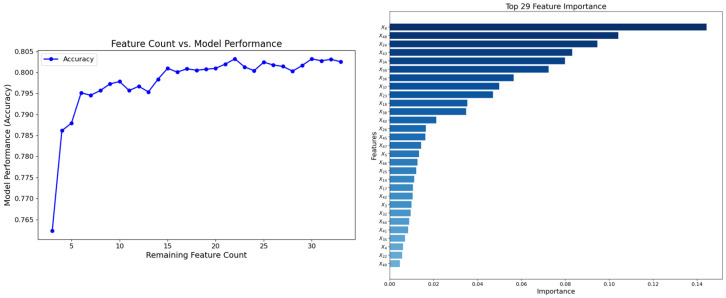
XGBoost RFE iteration trends and feature importance ranking.

Figure 3 illustrates the relationship between the number of remaining features and the model’s performance during the RFE process, along with the importance score ranking of the 29 features. Among these, feature $X_{8}$ achieved the highest score, while feature $X_{49}$ ranked the lowest. This approach not only reduced redundancy but also improved the predictive efficiency and interpretability of the model.

The correlation heatmap and MI analysis results show that the 29 retained features exhibit low inter-feature correlations, indicating that the redundancy was effectively eliminated while preserving those with a significant discriminatory power for Y. Furthermore, the bar chart provides a visual representation of the information gain contributed by each feature, further validating the explanatory power of the selected features in relation to the target. These results demonstrate that the proposed method removed irrelevant and redundant features, ensuring the robustness of the final feature set.

To validate the effectiveness of the two-stage algorithm proposed in this study, experiments were conducted using three classifiers: ANN, GBDT, and SVM. Four datasets were selected for comparison: datasets processed by the ReliefF algorithm (Z. Huang et al., 2018), the PSO algorithm (P. Wang & Wang, 2024), the proposed two-stage algorithm, and the original dataset without feature selection. During the experiments, five-fold cross-validation and parameter optimization were employed to obtain the optimal hyperparameter configurations. The detailed settings are shown in Table A1 (see Appendix A).

By conducting comparative experiments on the four datasets, the results for the three models—ANN, GBDT, and SVM—on metrics such as Acc, F1, AUC, and the feature dimensions were obtained. The detailed results are presented in Table 4.

**Figure 4:**
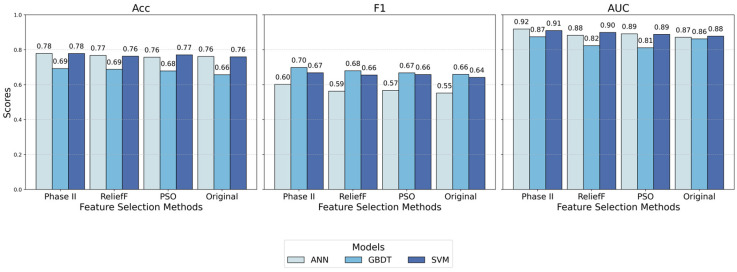
Comparison of feature selection algorithms in different models.

To visually demonstrate the performance differences of the various feature selection algorithms across models, a comparison chart was created, as shown in Figure 4.

From the perspective of classifiers, the ANN model performs best in terms of Acc and AUC, effectively capturing the non-linear relationships within the data. SVM follows, offering stable precision across different datasets. Although GBDT lags slightly behind the other two models, it stands out in terms of F1, indicating better performance on imbalanced datasets. This is because F1, as a harmonic mean of P and R, more accurately reflects the classifier’s performance on minority class samples.

From the perspective of feature selection algorithms, the proposed two-stage algorithm achieves the best performance across all classifiers, particularly in ANN and SVM, where it achieves higher Acc and AUC values. This further validates the proposed algorithm’s effectiveness in removing redundancy. In comparison, ReliefF retains too many features, which somewhat improves accuracy but fails to balance maximizing model performance and minimizing feature count. Meanwhile, although PSO selects fewer features, its performance in terms of Acc is slightly inferior, highlighting the trade-off between feature quantity and quality in selection processes. Using the original features directly yields the worst results, indicating the presence of redundancy and irrelevance in the raw data, underscoring the necessity of feature selection. Additionally, using only Stage 2 for feature selection resulted in a higher feature dimension and lower model accuracy compared to the proposed two-stage algorithm.

Additionally, Table A2 (see Appendix A) presents the prior probability for variable $X_{3}$ under different states of the target variable. These data preliminarily reveal the distribution characteristics of the variable, including its frequency, distribution proportions across each state, central tendency, and dispersion. These insights provide valuable reference points for subsequent causal relationship analyses and model optimization.

## 5. Educational Satisfaction Evaluation System

### 5.1. Tree Augmented Naive Bayesian Network

Based on the selected features, NB and TAN were further employed in this study to model and uncover the causal relationships within educational satisfaction, using overall satisfaction as the target variable to analyze the influence of satisfaction-related features under both frameworks. The dataset was split into training and testing sets in a 6:4 ratio, with 5341 samples used for training and 3562 samples used for testing.

First, overall satisfaction was set as the sole parent node. From Y, 29 directed edges were established, each pointing to one of the 29 attribute variables, thereby forming the structure of an NB network. The model learned the conditional probability distribution of each attribute variable using the training set and subsequently evaluated its performance on the testing set. The confusion matrix is shown in Table A3 (see Appendix A), providing insights into the model’s reliability and classification accuracy.

**Figure 5:**
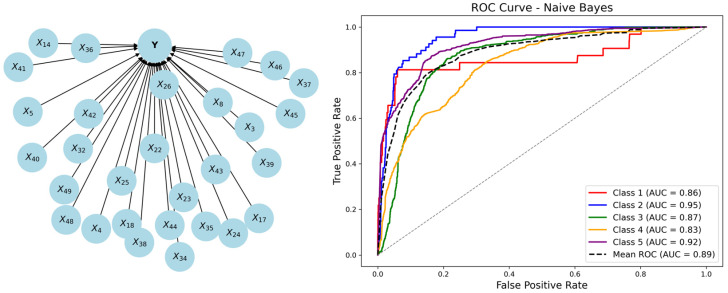
NB network structure and ROC curves.

When the posterior probability threshold was set to 0.5, the overall accuracy of the NB model reached 68.48%. Figure 5 illustrates the NB network structure and its corresponding ROC curves, with the model’s AUC value reaching 88.1%. This indicates a reasonable level of overall classification performance.

However, NB assumes that all attribute variables are independent, an assumption that does not always hold true in practical satisfaction evaluations. To address this limitation, we introduced the TAN model, which fully accounts for mutual information among attribute variables. Compared to NB, the TAN algorithm incorporates associative edges, allowing it to model dependencies more effectively.

**Figure 6:**
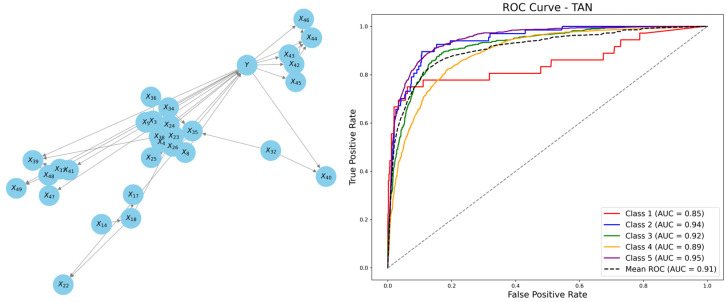
TAN network structure and ROC curves.

Figure 6 illustrates the TAN network structure along with its corresponding ROC curves. The AUC value reached 91.01%, significantly outperforming the NB.

In the TAN network, the overall satisfaction Y remains the sole parent node. However, beyond the directed edges extending from Y to the 29 attribute variables, TAN introduces associative edges between them, which reflect the correlations, resulting in a more complex network structure. For instance, the network topology reveals that $X_{32}$ (subject specialization) influences $X_{35}$ (independent academic research ability) and $X_{40}$ (multi-disciplinary knowledge), indicating that the value of $X_{32}$ affects the conditional probabilities of $X_{35}$ and $X_{40}$. Such dependency relationships uncover the latent interactions between variables, providing more intuitive support for causal analysis.

Table 5 presents the confusion matrix for the TAN model. When the posterior probability threshold was set to 0.5, the model achieved an overall Acc of 78.64%. Specifically, the Acc for predicting the highest satisfaction category (Y = 5) reached 86.33%, while the Acc for the lowest category (Y = 1) improved to 60%.

The introduction of the TAN model not only achieved a higher classification accuracy but also offered deeper insights into the underlying causal relationships among variables. Compared to the NB model, TAN showed significant improvements in overall accuracy and reliability, further validating the positive impact of incorporating variable interdependencies on predictive performance.

### 5.2. Causal Model Comparison Experiment

To validate the effectiveness of Bayesian networks in predicting educational satisfaction, three commonly used machine learning algorithms—ANN, GBDT, and SVM—were applied to the same dataset, and their performance was compared with the NB and TAN models. The dataset division and the proportion of the training and testing sets followed the same configuration as the previous process.

Table 6 presents the performance of the five models on the test set. The TAN demonstrated superior performance across all evaluation metrics (Acc, F1, and AUC), significantly outperforming the other models. This result further confirms the overall advantage, indicating that the TAN model not only achieves a high classification accuracy but also effectively captures the latent dependencies among variables.

**Figure 7:**
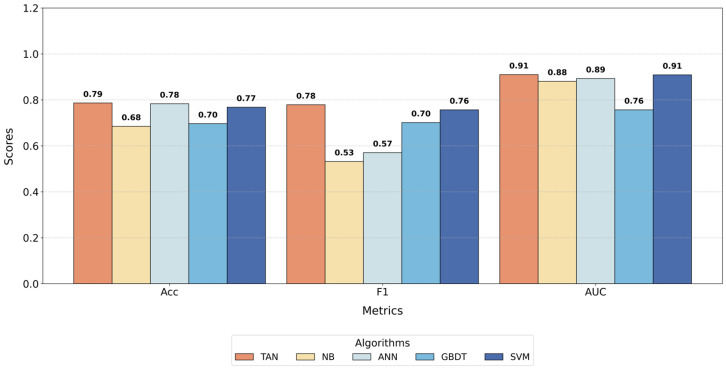
Comparison chart of different metrics across different classifiers.

To provide a more intuitive comparison of the classification performance of each model, the experimental results are visualized in Figure 7.

The analysis reveals that the TAN model outperformed all other models across all metrics. From the perspectives of feature selection and causal analysis, the superior performance of the TAN lies in its ability to account for correlations between features, enabling it to capture potential dependencies among variables more effectively than other models. Among traditional models, ANN achieves high Acc and AUC values but exhibits lower F1, indicating limited capability in handling multi-class balance issues. While SVM’s is close to TAN’s, its overall classification Acc is slightly inferior. Additionally, GBDT excels in F1, reflecting its strength in handling imbalanced data, but its low AUC value highlights its limited ability to differentiate between positive and negative samples.

**Figure 8:**
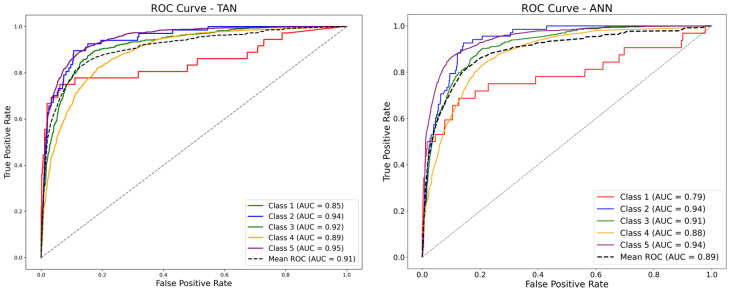

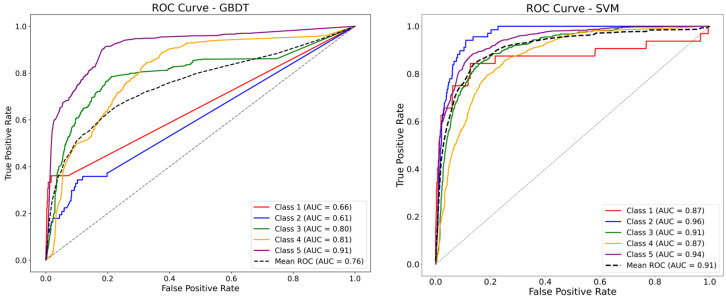
ROC curves for four classifiers.

Figure 8 further illustrates the ROC curves of the four models. It is evident that the TAN model’s curve consistently surpasses those of the other models, particularly in the low false positive rate region, demonstrating its significant advantage in identifying positive samples. In contrast, the GBDT model’s curve is noticeably lower than the others. The ROC curves of SVM and ANN are relatively close but still exhibit a clear gap compared to TAN, highlighting TAN’s superior performance.

In summary, the TAN model not only demonstrates exceptional performance but also effectively leverages the correlations among variables. This advantage enables this model to stand out in the task of predicting satisfaction, showcasing its robust capability in multi-class classification problems.

### 5.3. Importance Ranking of Influencing Factors

This experiment combined causal inference through Bayesian networks with the Birnbaum importance metric to quantitatively rank the key factors influencing satisfaction. By using Y = 5 (indicating high satisfaction) as the reliability metric for prediction, the prior and posterior probabilities of the attribute variable under different states were calculated to quantitatively analyze the impact of each feature on the target. The results not only reveal the contribution of each feature to changes in satisfaction but also provide scientific evidence for improving education quality and optimizing enrollment strategies.

The study assessed the impact of 29 features and ranked them based on Birnbaum importance. Table 7 presents the top six features, along with their corresponding prior probabilities, posterior probabilities, and importance values. The experimental results indicate that academic resilience ($X_{39}$), academic aspirations ($X_{37}$), dedication and service spirit ($X_{48}$), creative ability ($X_{34}$), academic standards ($X_{38}$), and independent academic research ability ($X_{35}$) are the top six critical factors influencing changes in satisfaction.

By analyzing the top-ranked features, it was found that the dimensions of academic enhancement, quality development, and mentor’s role contribute most significantly to educational satisfaction.

Academic enhancement is the core objective of graduate education, including indicators such as academic resilience ($X_{39}$), academic aspirations ($X_{37}$), and academic standards ($X_{38}$). These top-ranked features highlight the fact that students prioritize the development of their academic capabilities, reflecting a strong desire to achieve breakthroughs in academia through graduate education. Notably, academic resilience ranked first, with an importance score of 0.39115, emphasizing its critical role in shaping satisfaction.

Quality development, represented by dedication and service spirit ($X_{48}$) and creative ability ($X_{34}$), also emerged as being highly significant. $X_{48}$ ranked third with an importance score of 0.38979, further highlighting the critical role of postgraduate education in nurturing students’ overall competencies, especially in areas such as social responsibility. Mentor’s role is prominently reflected in the cultivation of academic interest ($X_{18}$), underscoring the pivotal role of mentors in guiding students to conduct independent academic research and improve logical thinking skills.

**Figure 9:**
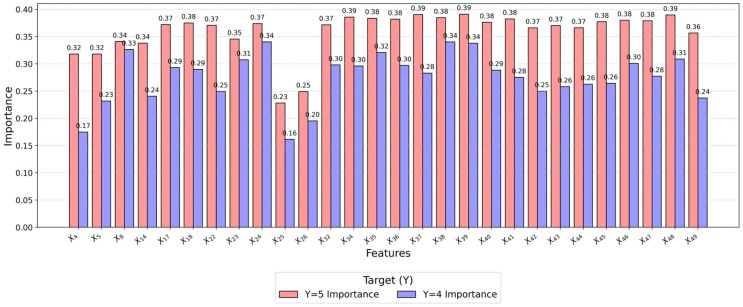
Importance of influencing factors in Y=5 vs. Y=4 states.

As shown in Figure 9, the importance levels of influencing factors under Y = 5 (high satisfaction) and Y = 4 (moderate satisfaction) states were compared. Significant differences were observed in the impact of various features on the target variable, especially under high satisfaction states, where certain features demonstrated particularly strong contributions. Taking $X_{39}$ as an example, its posterior probability exhibited fluctuations across different states, indicating high sensitivity. When $X_{39}$ was in state 5, the posterior probability of Y = 5 reached 0.74419, which was substantially higher than other states.

The state transition trends reveal that for most key features, as the state transitions from low to high scores, the posterior probability of the target variable shows a significant upward trend. For instance, the posterior probability of $X_{39}$ gradually increases from state 1 to state 5, reflecting that achieving a high level of academic resilience is critical for enhancing satisfaction.

### 5.4. Optimal Strategy Identification for Satisfaction Factors

Compared to merely predicting satisfaction levels, optimizing the state combinations of key indicators offers a more effective approach to enhancing satisfaction. However, traditional methods, while providing general directions, often lack specific and actionable recommendations. To address this gap, this study introduces a combination search-based optimization strategy to identify the optimal state combinations for improving educational satisfaction, offering evidence-based guidance for educational improvements.

This research involved a comprehensive analysis of all possible state combinations of 29 key indicators, and the posterior probability of each combination was calculated for Y = 5. With each indicator containing five possible states, the theoretical search space comprises $5^{29}$ possible state combinations. Given this massive computational space, an efficient algorithm was employed to calculate and filter systematically.

The optimization goal was set as a posterior probability of no less than 95% ($P\left(Y=5\right)$ ≥ 95%), and combinations meeting this criterion were ranked in descending order.

Table 8 presents the recommended state combinations for Y = 5 (partial results). Among all qualifying combinations, 722 state combinations met the criterion. For clarity, the top eight combinations with the highest posterior probabilities are shown. This method ensures that actionable insights are derived from the data, bridging the gap between theoretical modeling and practical implementation. By identifying precise strategies for improving satisfaction, it provides valuable guidance for improving postgraduate education quality and enhancing overall satisfaction levels.

Based on the recommendations in Table 8, the optimal combination to achieve the highest satisfaction level corresponds to a specific state configuration, which results in a posterior probability of 99.961%. This demonstrates that optimizing the 29 key indicators can significantly elevate educational satisfaction to near-perfect levels.

Further analysis reveals the following key insights:

(1) Characteristics of high posterior probability combinations: It is evident that most features in the recommended combinations are in high-level-score states. These states reflect students’ prioritization of excellence in academic support, which highlights the critical areas where education administrators should focus their improvement efforts.
(2) Priority of key indicators: In the top eight recommended combinations, certain indicators exhibit consistent high states (e.g., $X_{8}$ and $X_{37}$ remain in state 5), while others, such as $X_{17}$, show more variability in state configurations. This suggests that certain indicators play a more significant role in achieving high satisfaction levels. For instance, consistent high states in indicators underscore their pivotal role in driving satisfaction.
(3) Practicality of optimization strategies: These recommended combinations provide actionable insights for educational administrators. For example, maintaining the state of academic resilience ($X_{39}$) at a high level is critical, while indicators related to the mentor’s role and quality development (e.g., $X_{8},X_{37}$) also require focused attention.

These findings offer a clear framework for enhancing the educational experience for postgraduates. By focusing on maintaining high-level states in critical indicators and prioritizing resources toward these areas, administrators can systematically improve student satisfaction, ultimately contributing to the overall quality of postgraduate education.

## 6. Discussion

In this study, we introduce an innovative research methodology integrating Bayesian networks with feature importance analysis to quantitatively assess how behavioral and contextual factors interact to shape postgraduate education satisfaction. This approach identifies key indicators and clarifies their specific impact on satisfaction. The research is based on rigorous data from 8903 valid questionnaires, employing statistical validation methods, including CFA, to ensure reliability and scientific rigor. A posterior probability target combination search strategy, with a threshold of at least 90%, identifies the optimal combination of key indicators, offering actionable optimization suggestions.

Theoretically and practically, this research fills the gap in studying postgraduate education satisfaction by applying causal modeling and feature quantification, offering a systematic analytical framework for multi-indicator, multi-state complex systems. By integrating Bayesian networks with Birnbaum’s importance theory, it comprehensively analyzes the impact of various indicators on satisfaction. The findings highlight academic enhancement, quality improvement, and the mentor’s role as core dimensions, with key behavioral indicators such as academic resilience and academic aspirations.

From an educational practice perspective, postgraduates’ core expectations center on the improvement of academic abilities and comprehensive qualities. The primary goal of postgraduate education is to cultivate students’ academic innovation and research capabilities. Academic resilience directly influences students’ evaluations. The importance of academic aspirations and commitment to service suggests that students value the realization of intrinsic goals alongside their broader responsibilities to society. The significant impact of the mentor’s role highlights the critical position of faculty guidance, serving as transmitters of academic knowledge and key providers of career development.

Compared to previous studies, this research enriches the methods of quantitative analysis for educational satisfaction. Traditional studies often rely on regression analysis or analytic hierarchy processes, focusing primarily on the direct effects of individual indicators while overlooking the interdependencies among them. This study addresses this gap by employing TAN to comprehensively model the behavioral and cognitive mechanisms underlying student satisfaction, revealing how students’ engagement patterns, perceived support, and learning motivations interact to shape their overall experience. The integration of the Birnbaum importance not only quantitatively evaluates the importance of individuals but also identifies optimized strategies through state combination searches, providing a novel pathway for precise enhancement.

## 7. Conclusions

Based on the findings, the following management recommendations are proposed:

(1) Focus on academic quality development: Universities should optimize course designs and provide more opportunities for research training that encourage active learning behaviors, foster academic resilience, and enhance intrinsic motivation. This can be achieved through structured mentorship programs, stress management workshops, and resilience-building curricula, enabling students to develop sustained engagement.
(2) Enhance the role of mentorship: Universities should prioritize the recruitment and training of mentors, emphasizing their role in shaping students’ learning behaviors, career decision-making, and psychological well-being. In particular, mentorship programs should be designed to strengthen academic resilience and provide support for students in overcoming academic challenges.
(3) Promote comprehensive quality education: Institutions should diversify course offerings and incorporate behavioral interventions to foster collaborative learning and real-world engagement, ultimately strengthening students’ sense of social responsibility. Strengthening academic aspirations and service spirit can be facilitated through public service fellowships and career planning support.
(4) Institutionalize support for creative and independent research: Universities should expand research assistantship opportunities and provide interdisciplinary project grants, supporting students’ development of independent research skills.

This study has certain areas for improvement, which future research can address:

(1) Sample size and diversity: Future research can incorporate samples from diverse regions and disciplines to further validate the model’ s applicability and generalizability.
(2) Inclusion of new features: Exploring psychological factors related to satisfaction, such as learning stress and sense of belonging, could enrich the evaluation framework.
(3) Algorithm and model improvement: Incorporating advanced algorithms such as deep learning or more efficient combinatorial search methods could enhance the model’ s computational efficiency in handling ultra-large-scale data and improve its ability to analyze nonlinear relationships among features.

In summary, this study integrated behavioral insights with causal modeling to offer a novel approach to educational satisfaction evaluation. We identified key satisfaction drivers and provided actionable strategies to enhance educational quality. The findings serve as a valuable reference for policy management, supporting the development of targeted, behaviorally informed educational improvements.

## Figures and Tables

**Table 1:** TAN modeling process.

Input Dataset: Acquire the training dataset D.
1. Calculate Conditional Mutual Information: $I_{P_{D}}\left(A_{i};A_{j}|C\right),$ where i≠j.
2. Construct a Complete Undirected Graph: Each feature $A_{1},A_{2},\ldots ,A_{n}$ corresponds to a node, and the weight of the edge between any two features is determined by their conditional MI.
3. Build the Maximum Weight Spanning Tree.
4. Select a Root Attribute and Orient the Tree.
5. Add the Class Node: Add a node C to the directed tree, and introduce an arc to each $A_{i}$.
6. Construct the TAN Model.

**Table 2:** Postgraduate education satisfaction indicator system.

Dimension	Indicator	Variable
Course Quality	Teaching Quality	$X_{1}$
	Course Difficulty	$X_{2}$
	Enhancement of Ideology and Morality	$X_{3}$
	Enriching Humanistic Qualities	$X_{4}$
	Strengthen Professional Knowledge	$X_{5}$
	Understand the Frontiers of Science	$X_{6}$
	Learning Research Methods	$X_{7}$
Research Projects	Difficulty of Research Tasks	$X_{8}$
	Number of Research Projects	$X_{9}$
	Enhancement of Research Capacity	$X_{10}$
Mentor Guidance	Political Quality	$X_{11}$
	Teacher Ethics	$X_{12}$
	Mentoring Ability	$X_{13}$
	Mentoring Frequency	$X_{14}$
	Academic Level	$X_{15}$
	Practical Ability	$X_{16}$
Mentor’s Role	Cultivate Ideal Beliefs	$X_{17}$
	Stimulate Academic Interest	$X_{18}$
	Enhancement of Research Ability	$X_{19}$
	Correcting the Attitude of Scholarship	$X_{20}$
	Comply with Academic Standards	$X_{21}$
	Clarify Career Planning	$X_{22}$
Faculty Management	Faculty Service Evaluation	$X_{23}$
	Evaluation of Faculty Atmosphere	$X_{24}$
	Scholarship System	$X_{25}$
	Three-assistant Positions	$X_{26}$
	Library	$X_{27}$
	Cafeteria	$X_{28}$
	Accommodation	$X_{29}$
	Mental Health Counseling	$X_{30}$
	Career Guidance and Services	$X_{31}$
Academic Enhancement	Subject Specialization	$X_{32}$
	Professional Skills	$X_{33}$
	Creative Ability	$X_{34}$
	Independent Academic Research Ability	$X_{35}$
	Academic Writing Ability	$X_{36}$
	Academic Aspirations	$X_{37}$
	Academic Standards	$X_{38}$
	Academic Resilience	$X_{39}$
Quality Development	Multi-disciplinary Knowledge	$X_{40}$
	Willingness to Serve the Country	$X_{41}$
	Interpersonal Skills	$X_{42}$
	Public Speaking Skills	$X_{43}$
	Organizational and Leadership Skills	$X_{44}$
	Time Management Skills	$X_{45}$
	Teamwork Ability	$X_{46}$
	Ability to Understand National Conditions	$X_{47}$
	Dedication and Service Spirit	$X_{48}$
	International Exchange Ability	$X_{49}$
Overall Evaluation	Overall Educational Satisfaction	Y

Note: $X_{11}$ refers to the mentor’s ability to provide ideological and ethical leadership. $X_{20}$ indicates guidance on ethical research. $X_{26}$ refers to roles such as teaching, research, and administrative assistants, offering financial support and practical experience.

**Table 3:** Reliability and validity testing results of the scale.

Dimension	Variable	Cronbach’s α	CR	AVE	KMO	Bartlett $\chi^{2}$	p-Value
Course Quality	$X_{1}$–$X_{7}$	0.9096	0.862	0.660	0.902	1314.00	1.01 × 10^−280^
Research Projects	$X_{8}–X_{10}$	0.7924	0.679	0.504	0.514	3716.64	0
Mentor Guidance	$X_{11}–X_{16}$	0.9795	0.857	0.891	0.927	176.82	2.560 × 10^−36^
Mentor’s Role	$X_{17}–X_{22}$	0.9800	0.857	0.893	0.923	225.58	9.491 × 10^−47^
Faculty Management	$X_{23}–X_{31}$	0.9281	0.895	0.611	0.908	2411.89	0
Academic Enhancement	$X_{32}–X_{39}$	0.9709	0.888	0.815	0.949	548.94	2.38 × 10^−114^
Quality Development	$X_{40}–X_{49}$	0.9772	0.909	0.813	0.965	367.60	1.111 × 10^−73^
Overall Scale	$X_{1}–X_{49}$	0.9755	0.978	0.519	0.982	23,698.65	0

**Table 4:** Comparative experimental results of feature selection algorithms.

Dataset	Model	Acc	F1	AUC	Feature Dimensions
Phase II	ANN	0.779062	0.601817	0.918843	29
	GBDT	0.692632	0.698461	0.874274	
	SVM	0.778614	0.668241	0.909957	
ReliefF	ANN	0.767546	0.592902	0.882820	40
	GBDT	0.687760	0.679612	0.863432	
	SVM	0.763161	0.655332	0.899135	
PSO	ANN	0.757148	0.567243	0.891128	20
	GBDT	0.678664	0.667887	0.811622	
	SVM	0.770915	0.658075	0.887956	
Original	ANN	0.761651	0.552087	0.871393	49
	GBDT	0.657198	0.659140	0.862437	
	SVM	0.759512	0.641684	0.877895	

**Table 5:** Confusion matrix for TAN model.

	1 (15)	2 (29)	3 (390)	4 (1825)	5 (1303)
Confusion Matrix					
1 (36)	9	9	8	4	6
2 (67)	2	12	41	12	0
3 (498)	4	7	273	196	18
4 (1732)	0	1	67	1446	218
5 (1229)	0	0	1	167	1061
Reliability					
1 (36)	60.00%	31.03%	2.05%	0.22%	0.46%
2 (67)	13.33%	41.38%	10.51%	0.66%	0.00%
3 (498)	26.67%	24.14%	70.00%	10.74%	1.38%
4 (1732)	0.00%	3.45%	17.18%	79.23%	16.73%
5 (1229)	0.00%	0.00%	0.26%	9.15%	81.43%
Accuracy					
1 (36)	25.00%	25.00%	22.22%	11.11%	16.67%
2 (67)	2.99%	17.91%	61.19%	17.91%	0.00%
3 (498)	0.80%	1.41%	54.82%	39.36%	3.61%
4 (1732)	0.00%	0.06%	3.87%	83.49%	12.59%
5 (1229)	0.00%	0.00%	0.08%	13.59%	86.33%

**Table 6:** Experimental results comparing different classifiers.

Classifier	*Acc*	*F*1	*AUC*
TAN	0.786356	0.778732	0.910053
NB	0.684762	0.531785	0.881002
ANN	0.782987	0.570426	0.892807
GBDT	0.696519	0.701328	0.756751
SVM	0.768108	0.756780	0.908908

**Table 7:** Importance ranking of influencing factors (top six).

Variable	State	Prior Probability	Posterior Probability	Importance	Ranking
$X_{39}$	1	0.00831	0.02703	0.39115	1
	2	0.02853	0.03150		
	3	0.14265	0.02913		
	4	0.44333	0.15201		
	5	0.37718	0.74419		
$X_{37}$	1	0.01820	0.02469	0.39047	2
	2	0.03527	0.02548		
	3	0.15781	0.04484		
	4	0.42997	0.16588		
	5	0.35876	0.76268		
$X_{48}$	1	0.00809	0.02778	0.38979	3
	2	0.03684	0.03963		
	3	0.14613	0.03228		
	4	0.43850	0.15471		
	5	0.37044	0.75349		
$X_{34}$	1	0.00753	0.04478	0.38579	4
	2	0.03830	0.01760		
	3	0.17837	0.05542		
	4	0.43839	0.18012		
	5	0.33741	0.78096		
$X_{38}$	1	0.00562	0.06000	0.38479	5
	2	0.02718	0.02479		
	3	0.13726	0.03110		
	4	0.45165	0.14822		
	5	0.37830	0.74317		
$X_{35}$	1	0.00517	0.04348	0.38377	6
	2	0.02527	0.02222		
	3	0.14557	0.04861		
	4	0.45928	0.15847		
	5	0.36471	0.74777		

**Table 8:** Recommended table of indicator combinations under Y=5(P≥ 95%).

P(Y=5)	99.961%	99.960%	99.959%	99.946%	99.929%	99.929%	99.929%	99.919%
$X_{3}$	2	3	3	4	1	3	3	3
$X_{4}$	2	4	1	2	2	4	1	5
$X_{5}$	5	3	3	4	5	2	4	1
$X_{8}$	4	5	5	5	5	5	5	5
$X_{14}$	3	5	3	3	5	4	5	2
$X_{17}$	2	4	4	2	1	5	3	3
$X_{18}$	2	1	2	2	2	1	1	5
$X_{22}$	5	5	5	5	5	5	5	5
$X_{23}$	5	5	3	3	5	5	5	2
$X_{24}$	5	5	2	2	5	5	5	5
$X_{25}$	5	1	1	5	5	5	5	5
$X_{26}$	2	5	3	5	2	1	4	1
$X_{32}$	5	3	2	2	3	2	5	3
$X_{34}$	3	4	5	4	1	1	1	4
$X_{35}$	1	2	4	2	2	2	4	1
$X_{36}$	5	5	3	5	2	5	4	5
$X_{37}$	5	5	5	5	5	5	5	5
$X_{38}$	5	5	2	1	4	4	2	2
$X_{39}$	3	3	5	5	1	5	4	5
$X_{40}$	1	3	3	2	1	5	1	4
$X_{41}$	1	2	2	2	3	5	3	1
$X_{42}$	2	1	4	5	4	1	3	3
$X_{43}$	1	4	1	3	1	4	4	4
$X_{44}$	1	2	1	4	3	1	3	3
$X_{45}$	4	3	3	2	1	1	3	1
$X_{46}$	5	2	2	1	2	5	4	4
$X_{47}$	5	2	1	5	1	4	1	4
$X_{48}$	2	1	2	4	1	1	3	4
$X_{49}$	2	3	4	2	2	2	5	2

## Data Availability

The experimental data used to support the findings of this study are available from the corresponding author upon request.

## Appendix A

**Table A1:** Optimal hyperparameter settings for each algorithm.

Algorithm	Parameter	Setting Value
GBDT	n_estimators	150
	max_depth	3
	learning_rate	0.01
SVM	C	0.1
	gamma	scale
	kernel	linear
ANN	hidden_layer_sizes	50,50
	activation	relu
	solver	adam
PSO	population_size	50
	max_iterations	0.01
ReliefF	k	20

**Table A2:** Modeling prior probability table ($X_{3}$).

Feature Variable	State	Y State	Frequency	Percentage
$X_{3}$	1	1	26	32.50%
		2	24	14.20%
		3	33	2.68%
		4	59	1.38%
		5	41	1.30%
	2	1	5	6.25%
		2	18	10.65%
		3	78	6.34%
		4	59	1.38%
		5	6	0.19%
	3	1	16	20%
		2	74	43.79%
		3	561	45.61%
		4	644	15.05%
		5	73	2.32%
	4	1	17	21.25%
		2	43	25.44%
		3	410	33.33%
		4	2287	53.46%
		5	334	10.62%
	5	1	16	20%
		2	10	5.92%
		3	148	12.03%
		4	1229	28.73%
		5	2692	85.57%

**Table A3:** Confusion matrix for NB model.

	1 (54)	2 (112)	3 (594)	4 (1563)	5 (1239)
Confusion Matrix					
1 (32)	13	8	3	4	4
2 (68)	9	32	22	5	0
3 (492)	18	56	292	113	13
4 (1711)	7	15	268	1162	259
5 (1259)	7	1	9	279	963
Reliability					
1 (32)	24.07%	7.14%	0.51%	0.26%	0.32%
2 (68)	16.67%	28.57%	3.70%	0.32%	0.00%
3 (492)	33.33%	50.00%	49.16%	7.23%	1.05%
4 (1711)	12.96%	13.39%	45.12%	74.34%	20.90%
5 (1259)	12.96%	0.89%	1.52%	17.85%	77.72%
Accuracy					
1 (32)	40.63%	25.00%	9.38%	12.50%	12.50%
2 (68)	13.24%	47.06%	32.35%	7.35%	0.00%
3 (492)	3.66%	11.38%	59.35%	22.97%	2.64%
4 (1711)	0.41%	0.88%	15.66%	67.91%	15.14%
5 (1259)	0.56%	0.08%	0.71%	22.16%	76.49%
